# Pain trajectories over 12 months following conservative care consultation in patients with lumbar spinal stenosis

**DOI:** 10.1186/s13104-024-06840-6

**Published:** 2024-06-22

**Authors:** Rikke K. Jensen, Lisbeth Hartvigsen, Alice Kongsted

**Affiliations:** 1 Department of Sports Science and Clinical Biomechanics, Center for Muscle and Joint Health, University of Southern Denmark, Campusvej 55, 5230 Odense, Denmark; 2 Chiropractic Knowledge Hub, Campusvej 55, 5230 Odense M, Denmark; 3 Private Chiropractic Practice, Hartvigsen & Hein, Vestergade 11, 5000 Odense C, Denmark

**Keywords:** Lumbar spinal stenosis, Neurogenic claudication, Trajectories, Low back pain, Leg pain, Disability, Chiropractic

## Abstract

**Objective:**

To investigate symptom trajectories in chiropractic patients with lumbar spinal stenosis (LSS).

**Methods:**

Patients diagnosed with LSS were recruited from chiropractic clinics and self-reported questionnaires were collected at baseline and 1-year follow-up. Patients received weekly text messages about low back pain (LBP) and leg symptoms for 1 year. Group-based trajectory modelling was performed to identify symptom trajectory groups. The groups were compared based on patient characteristics, LBP and leg pain intensity, Oswestry Disability Index (ODI) and Zurich Claudication Questionnaire (ZCQ).

**Results:**

A total of 90 patients were included in the analysis. A three-group trajectory model was chosen: ‘improving’ (16%), ‘fluctuating/improving’ (30%), and ‘persistent’ (54%). The ‘persistent’ group had a higher proportion of women [71% (95% CI 57–82%)] than the ‘improving’ group 29% (95% CI 11–56%), and a higher ODI score at both baseline [34.2 (95% CI 29.7–38.8) vs. 22.8 (16.4–29.1)] and 1-year follow-up [28.1 (95% CI 23.2–33.0) vs. 4.8 (0.1–9.4)]. Similar differences were observed for ZCQ symptom and function scores.

**Conclusions:**

Pain symptoms in people with LSS followed distinctly different trajectories. Half of the sample had a pattern of consistently severe symptoms over a year, while the other half either improved rapidly or experienced fluctuating symptoms with some improvement.

**Supplementary Information:**

The online version contains supplementary material available at 10.1186/s13104-024-06840-6.

## Introduction

Lumbar spinal stenosis (LSS) is a degenerative spinal condition that primarily affects the elderly. It is one of the main reasons for spinal surgery in older adults [1]. Clinical guidelines emphasise the importance of conservative treatment options for LSS [2, 3]. However, there is limited evidence on the clinical or natural course of the disease in patients who do not undergo surgery. Conservative treatment has been suggested to improve 30–50% of patients with LSS, according to experts. However, it is important to note that these estimates are based on consensus [4].

Over the past 20 years, it has become widely accepted that non-specific low back pain (LBP) is often an episodic condition, and researchers have identified several common LBP trajectories [5–7]. As LSS and LBP share overlapping symptoms [8], it is possible that similar trajectories exist for LSS.

Therefore, the objective of this study was to investigate the trajectories of symptom occurrence and severity in patients with LSS consulting for conservative care.

## Main text

### Method

#### Setting and participants

This was an exploratory prospective cohort study with repeated outcome measures of patients diagnosed with LSS based on clinical assessment and enrolled in an LSS care programme at a chiropractic clinic in Denmark. Treatments offered as part of the care programme included information, manual therapy, and exercise. The chiropractic clinics recruiting patients were part of a collective agreement regulated by the national health authorities, which included increased partial reimbursement for a standardised chiropractic care package for LSS. Patients were recruited between July 2018 and March 2020.

Patients aged 18 years or older who reported symptoms consistent with neurogenic claudication were assessed for eligibility. Symptoms of neurogenic claudication included a history of pain or other symptoms such as paraesthesia or heaviness in the buttocks and/or legs, worsening of symptoms with walking and/or prolonged standing, and relief with sitting and/or bending forward. A clinical examination was performed to exclude red flags (e.g. severe neurological deficits) and to consider differential diagnoses (e.g. vascular claudication, hip osteoarthritis, trochanteric bursitis) [9]. In addition, patients had to agree to be included in a care package, be able to send and receive text messages on their mobile phone, and be able to speak, read and understand Danish.

#### Data collection and variables

Patients enrolled in the project completed a questionnaire at baseline and at 1-year follow-up via a link to an online questionnaire, which was emailed after initial contact and again 1 year later. Contact information, written consent and questionnaire data were collected and stored using the online system Research Electronic Data Capture (REDCap), hosted and supported by the Odense Patient data Explorative Network (OPEN).

Data were collected on age, sex, body mass index, duration of symptoms (over/under 12 weeks), comorbid pain (yes/no), number of comorbidities (none, 1–2, > 2), general health (single item on general self-rated health with five response options ranging from very good to very poor health), previous treatment (yes/no) and use of pain medication (none, prescription or over-the-counter). Outcomes assessed at 1 year were LBP intensity (0–10), leg pain intensity (0–10) and functional disability as measured by the Oswestry Disability Index (ODI) (0–100) and the Zurich Claudication Questionnaire (ZCQ) symptom score (1–5) and function score (1–4).

In addition, patients received 3 weekly text messages over 1 year with questions about the number of days in the past week with LBP (0–7 days), leg symptoms (0–7 days), and symptom intensity on a scale of 0 (none) to 10 (worst). An electronic text message tracking service automated the weekly distribution of text message questions.

#### Statistical methods

Patients were excluded from the analysis if they responded to fewer than 20 out of 52 text messages in 1 year. Responders and non-responders were compared on baseline demographics and characteristics. Group-based trajectory modelling was performed using the traj command in STATA. The final number of groups was determined based on the Bayesian Information Criterion (BIC), an adequate sample size in each group, an average posterior probability value ≥ 0.7, and the interpretability of the model. Plots were generated to illustrate the trajectory groups used in our models using the trajplot command. Identified trajectory groups were compared for baseline variables, presented as frequencies and means with confidence intervals (CI), and for pain and function at 1-year follow-up [10, 11]. Data management and analyses were performed using STATA 17 (Stata Corp LLC, Collage Station, USA).

### Results

Of the 127 patients included, 90 (71%) responded to at least 20 text messages. Compared to non-responders, responders were more likely to be female, to have longer pain duration, more comorbidities, and to have received previous treatment (data not shown). They had a mean age of 70 years (SD 8.7), ranging from 47 to 89 years, and 58% were female. Mean LBP intensity was 5.6 (SD 2.5) and leg pain intensity was 5.1 (SD 2.8). The ODI was 30 (SD 13.7), and the ZCQ symptom and function scores were 2.9 (SD 0.6) and 2.4 (SD 0.6), respectively.

**Fig. 1:**
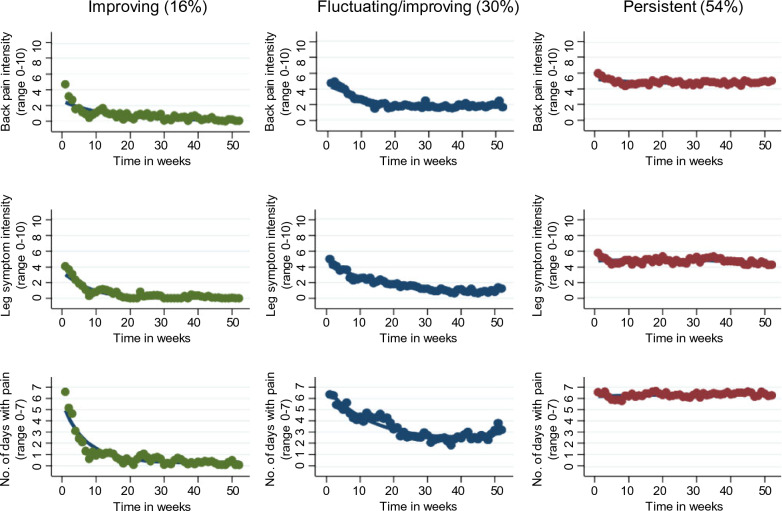
Trajectories for low back pain intensity, leg symptom intensity and number of days with pain by group

A three-group trajectory model was selected: ‘improving’ (16%), ‘fluctuating/improving’ (30%) and ‘persistent’ (54%) (Fig. 1). The mean posterior probability of being in each of the three groups was 100% (range 100% to 100%), 99.9% (range 99.9% to 100%) and 99.9% (range 99.9% to 100%) respectively. Individual plots by trajectory group are provided in Additional file 1.

The ‘persistent’ group had a higher proportion of women than the ‘improving’ group, but otherwise there were no major differences in patient demographics and characteristics (Table 1). However, the ‘persistent’ group appeared to be more severely affected in all observed parameters (e.g. pain duration, comorbidity, general health, use of health services and medication).

**Table 1:** Baseline characteristics and 1-year outcomes by group status

	Improving	Fluctuating/improving	Persistent
**Baseline**	14	27	49
Age, mean (95% CI) in years	68.1 (63.4–72.7)	68.2 (64.7–71.7)	70.6 (68.1–73.1)
Missing, n	0	0	0
Female, % (95 CI)	28.6 (11.0–56.4)	48.1 (30.2–66.6)	71.4 (57.1–82.4)
Missing, n	0	0	0
Body mass index, mean (95% CI) in kg/m^2^	26.1 (23.9–28.4)	27.7 (25.9–29.4)	28.0 (26.1–29.8)
Missing, n	0	0	3
Duration of leg symptoms, % (95% CI)			
< 12 weeks	41.7 (18.2–69.6)	40.0 (22.8–60.0)	25.5 (15.0–40.0)
> 12 weeks	58.3 (30.4–81.8)	60.0 (40.0–77.2)	74.5 (60.0–85.0)
Missing, n	2	2	2
Comorbid pain (yes), % (95% CI)	71.4 (43.5–89.0)	92.6 (74.4–98.2)	95.7 (84.3–99.0)
Missing, n	0	0	2
Number of comorbidities, % (95% CI)			
None	28.6 (11.0–56.4)	7.4 (1.8–25.6)	8.2 (3.1–20.0)
1–2	28.6 (11.0–56.4)	48.1 (30.2–66.6)	40.8 (27.9–55.1)
> 2	42.9 (20.4–68.7)	44.4 (27.0–63.3)	51.0 (37.1–64.8)
Missing, n	0	0	2
General health, n (%)			
Very good/good	85.7 (56.8–96.5)	74.1 (54.4–87.2)	46.8 (33.0–61.1)
Fair/poor/very poor	14.3 (3.5–43.2)	25.9 (12.8–45.6)	53.2 (38.9–67.0)
Missing, n	0	0	2
Previous treatment (yes), % (95% CI)	64.3 (37.3–84.5)	74.1 (54.4–87.2)	80.8 (66.9–89.8)
GP, % (95% CI)	28.6 (11.0–56.5)	40.7 (24.0–60.0)	48.9 (34.9–63.1)
Chiropractor, % (95% CI)	7.1 (1.0–37.7)	22.2 (10.2–41.8)	10.6 (4.4–23.4)
Physiotherapist, % (95% CI)	35.7 (15.5–62.7)	37.0 (21.0–56.5)	57.4 (42.9–70.8)
Missing, n	0	0	2
Current use of pain medication, % (95% CI)			
No	50.0 (25.7–74.3)	29.6 (15.4–49.3)	14.9 (7.2–28.3)
Prescription	14.3 (3.5–43.2)	25.9 (12.8–45.6)	57.4 (42.9–70.8)
Over-the-counter	35.7 (15.5–62.7)	44.4 (27.0–63.3)	27.7 (16.7–42.2)
Missing, n	0	0	2
Back pain intensity, mean (95% CI)	5.1 (3.8–6.4)	5.2 (4.3–6.2)	5.9 (5.1–6.6)
Missing, n	0	0	0
Leg pain intensity, mean (95% CI)	5.2 (3.6–6.8)	4.9 (3.8–6.0)	5.2 (4.4–6.0)
Missing, n	0	0	1
Oswestry Disability Index	22.8 (16.4–29.1)	26.0 (22.1–29.8)	34.2 (29.7–38.8)
Missing, n	0	0	4
Zurich claudication questionnaire			
Symptom score (1–5), mean (95% CI)	2.5 (2.2–2.7)	2.8 (2.6–3.0)	3.1 (3.0–3.3)
Missing, n	0	0	1
Function score (1–4), mean (95% CI)	1.9 (1.6–2.3)	2.3 (2.1–2.5)	2.6 (2.4–2.7)
Missing, n	0	0	1
**One-year follow-up**	14	27	49
Back pain intensity, mean (95% CI)	0.4 (0.0–0.8)	2.3 (1.5–3.1)	4.6 (3.7–5.6)
Missing, n	4	5	16
Leg pain intensity, mean (95% CI)	0.6 (0.0–1.5)	1.6 (0.9–2.3)	4.3 (3.4–5.3)
Missing, n	4	5	16
Oswestry Disability Index	4.8 (0.1–9.4)	15.2 (9.1–21.3)	28.1 (23.2–33.0)
Missing, n	6	9	17
Zurich claudication questionnaire			
Symptom score (1–5), mean (95% CI)	1.3 (1.0–1.5)	1.9 (1.7–2.1)	2.8 (2.6–3.0)
Missing, n	5	7	17
Function score (1–4), mean (95% CI)	1.2 (1.0–1.3)	1.5 (1.3–1.6)	2.0 (1.8–2.3)
Missing, n	6	7	17

At baseline, there were no differences in LBP or leg pain intensity. However, after 1 year, LBP and leg pain intensity were highest in the ‘persistent’ group compared with the ‘fluctuating/improving’ group and the ‘improving’ group. Patients in the ‘persistent’ group also had higher ODI scores at baseline than those in the ‘fluctuating/improving’ and ‘improving’ groups. This difference was more pronounced at the 1-year follow-up. At baseline, patients in the ‘improving’ group had lower scores on the ZCQ, indicating less severe symptoms and better function, compared with the ‘Persistent’ group. At the 1-year follow-up, there was a difference between all three groups, with the ‘improving’ group having the lowest scores, followed by the ‘fluctuating/improving’ group, and patients in the ‘persistent’ group having the highest scores.

### Discussion

In a sample of patients with LSS presenting for chiropractic care, three distinct pain trajectory patterns were identified. One group of patients showed improvement within the first 10–12 weeks (16%), another group had a fluctuating pain pattern with some improvement over time (30%) and the third group had more persistent symptoms (54%). The groups that showed improvement or had a fluctuating pattern with some improvement had considerably better outcome scores than the group with more persistent symptoms on disease-specific symptoms and function, which were not entered into the latent class analysis. To our knowledge, this is the first study to examine pain trajectory patterns in patients with LSS.

Based on ten studies with a total of almost 9000 patients, Kongsted et al. described simplified principal trajectory patterns of LBP. These patterns are similar to our findings in patients with LSS. In previous studies of LBP in primary care cohorts, about one in five patients were classified as having ‘severe patterns’, whereas about half of the patients with LSS belonged to this trajectory group. Apart from the difference in the number of trajectory groups and hence the difference in proportions, it is also important to consider that these findings apply specifically to LSS, which is considered to be a specific degenerative spinal disease, whereas LBP is multifactorial and includes short-term acute episodes [7, 9]. However, the misconception that LSS inevitably worsens over time cannot be supported as almost half of patients with LSS either improved or had fluctuating pain with some pattern of improvement.

### Limitations

The main limitation of this study is the small sample size, and the results should be interpreted with caution. The approach was exploratory and the small sample size did not allow for detailed analyses that might reveal rare but potentially important groups. It is therefore possible that future studies will identify different trajectories to our findings, although overall our results are consistent with previous findings in LBP populations. 90 patients responded to at least 20 text messages over the course of a year. However, between 28 and 36% of follow-up results were missing. Nevertheless, the differences in patient characteristics and outcomes identified between the groups provided some evidence that the groups identified were distinctly different.

Another limitation is the lack of diagnostic criteria for LSS. As with most other musculoskeletal conditions, the diagnosis of LSS is based on history and physical examination, which could lead to variability between patients included. MRI was not included in the diagnostic criteria because imaging is not necessary for initial assessment in primary care [12]. Although MRI can confirm the diagnosis of LSS, there is little correlation between patient symptoms and imaging findings, except in severe cases [13]. Finally, it is unclear what type of treatment patients received and for how long, which may have influenced both their trajectories and outcomes.

Visual interpretation of individual patient trajectories (Additional file 1) revealed some variation in patterns within each trajectory group. It is possible that the small sample size did not allow further differentiation and that a finer classification will emerge when these analyses are repeated in a larger study. However, there was a high level of confidence in the identification of the trajectories to which individuals were most closely matched.

### Conclusion

Patients with LSS showed distinctly different trajectories of pain symptoms. Half of the sample experienced severe symptoms throughout the year, while the remainder showed patterns of rapid improvement or fluctuating symptoms with some improvement. These findings reveal heterogeneous symptom trajectories among patients diagnosed with LSS. This variation in symptom progression in people with LSS suggests that LSS may not always present as a persistent or progressive condition. Further research with larger samples is needed to confirm these observations.

### Supplementary Information

Additional file 1. Individual pain trajectories stratified by trajectory group.

## Data Availability

Data are available upon reasonable request from the corresponding author.

## Abbreviations

BIC: Bayesian Information Criterion
CI: Confidence intervals
LBP: Low back pain
LSS: Lumbar spinal stenosis
OPEN: Odense Patient data Explorative Network
ODI: Oswestry Disability Index
REDCap: Research Electronic Data Capture
SD: Standard deviation
ZCQ: Zurich Claudication Questionnaire
