# Supplementary material for: Pain trajectories over 12 months following conservative care consultation in patients with lumbar spinal stenosis
Source: BMC Res Notes. 2024 Jun 22;17:174. doi: 10.1186/s13104-024-06840-6 (PMC11193895; doi:10.1186/s13104-024-06840-6)

Additional file 1

Individual pain trajectories stratified by trajectory group

The graphs show the trajectory of back pain, leg pain and number of days with pain measured each week for 52 weeks for each individual.

- Page 2 shows the trajectories for individuals belonging to a group with an overall improving pain pattern.
- Pages 3-4 show the trajectories for individuals with a fluctuating pattern with some improvement over time.
- Pages 5-8 show the trajectories for individuals with a more persistent pain pattern.

Group: Improving

Blue line: number of days with pain on a scale from 0 to 7

Red line: back pain intensity on a scale from 0 to 10

Green line: leg symptom intensity on a scale from 0 to 10

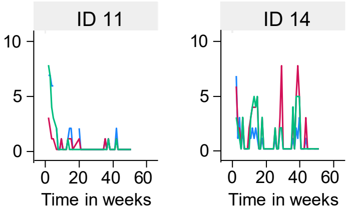

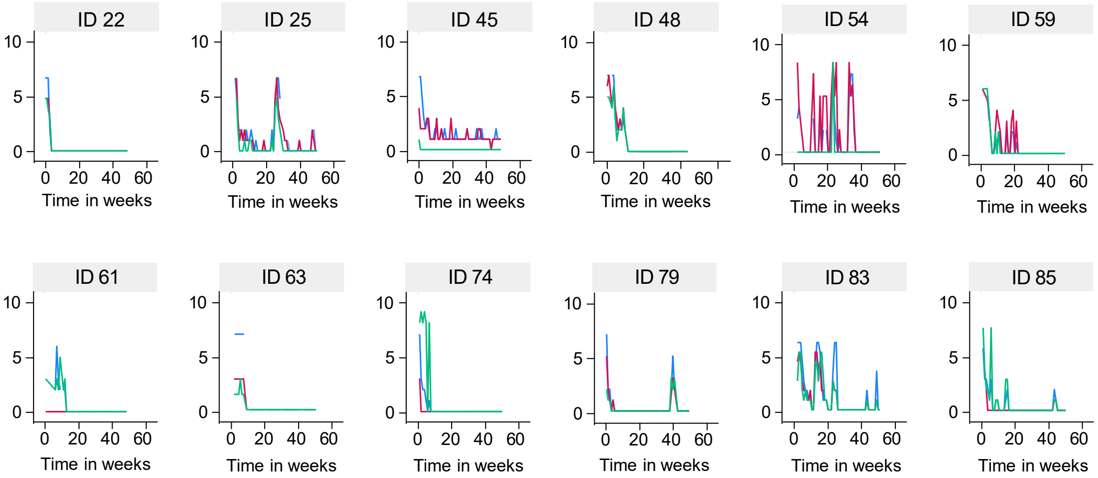

Group: Fluctuating/improving

Blue line: number of days with pain on a scale from 0 to 7

Red line: back pain intensity on a scale from 0 to 10

Green line: leg symptom intensity on a scale from 0 to 10

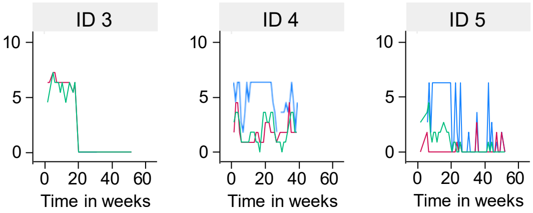

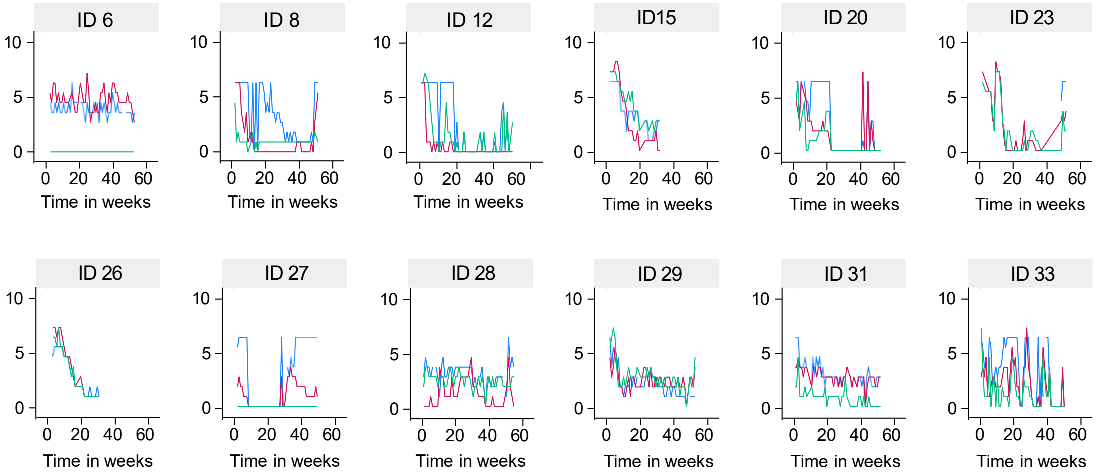

Group: Fluctuating/improving

Blue line: number of days with pain on a scale from 0 to 7

Red line: back pain intensity on a scale from 0 to 10

Green line: leg symptom intensity on a scale from 0 to 10

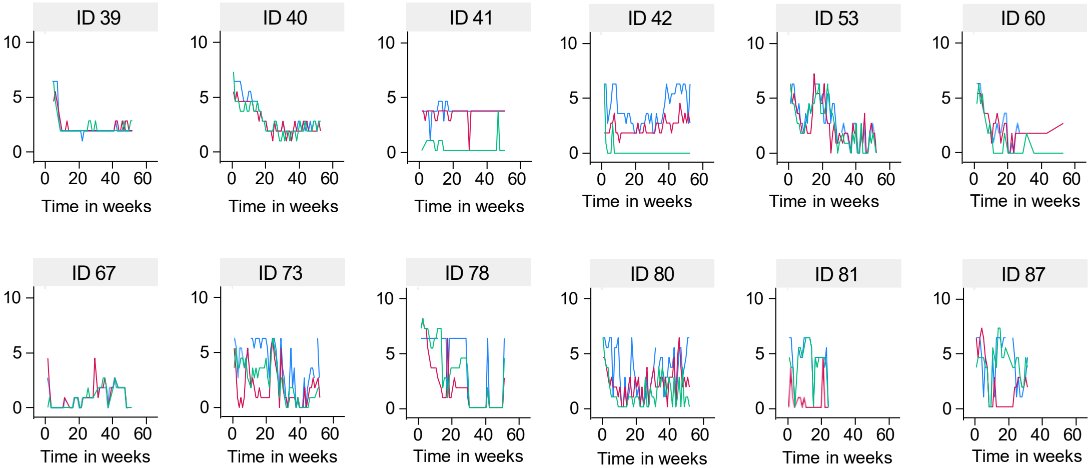

Group: Persistent

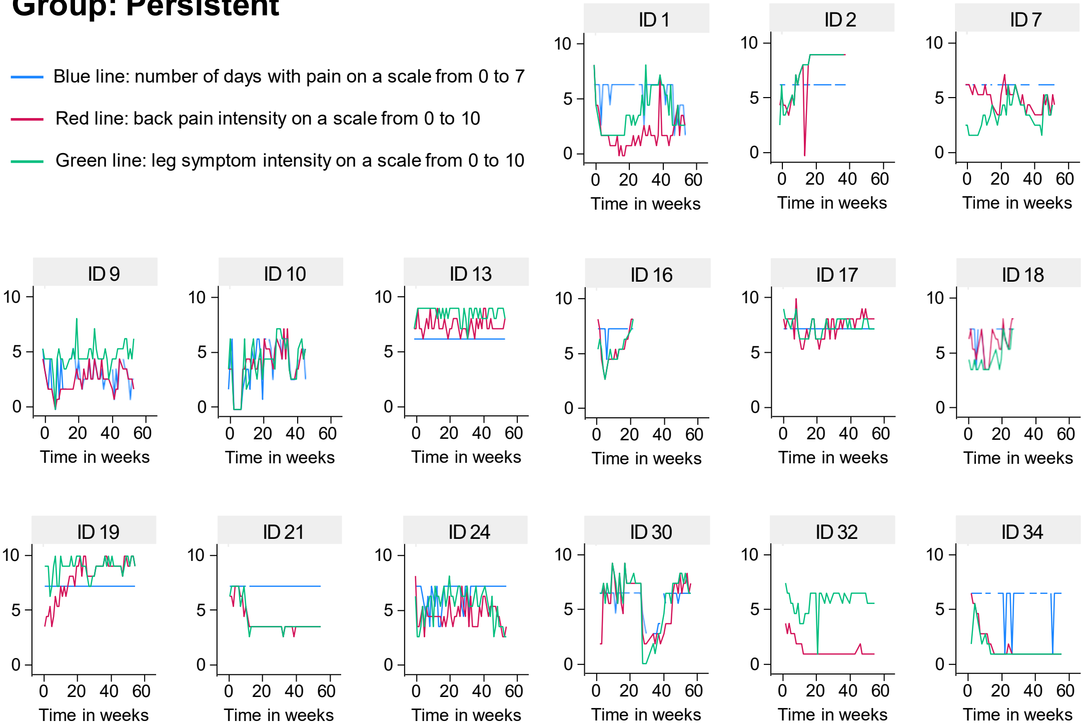

Group: Persistent

- Blue line: number of days with pain on a scale from 0 to 7
- Red line: back pain intensity on a scale from 0 to 10
- Green line: leg symptom intensity on a scale from 0 to 10

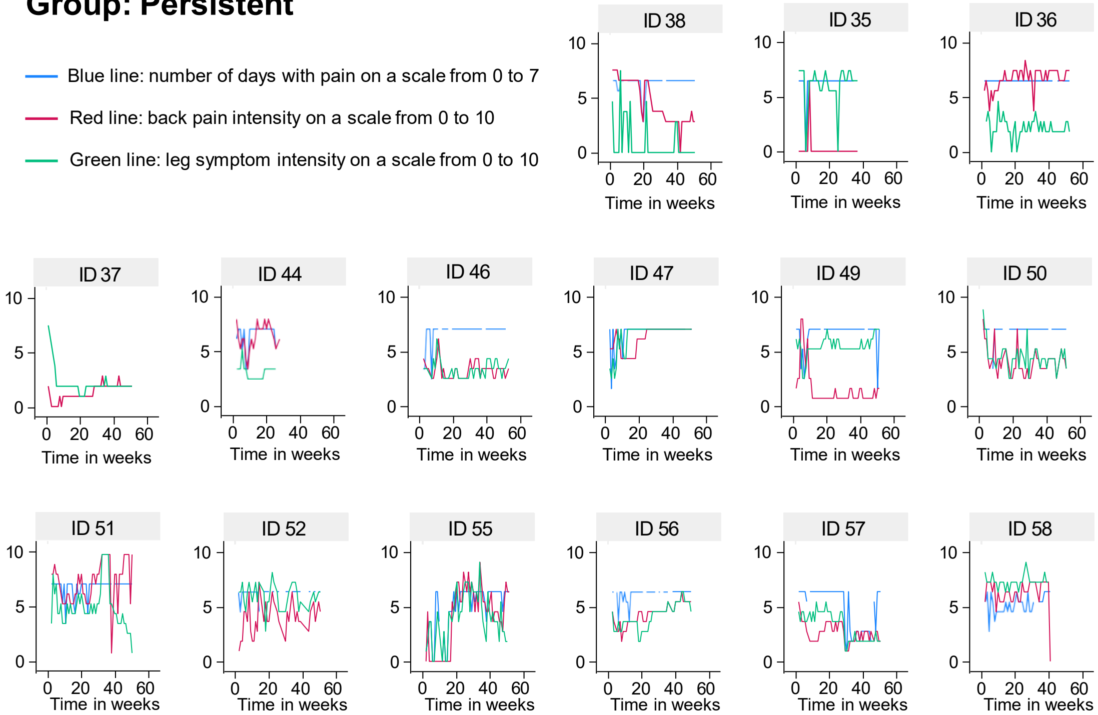

Group: Persistent

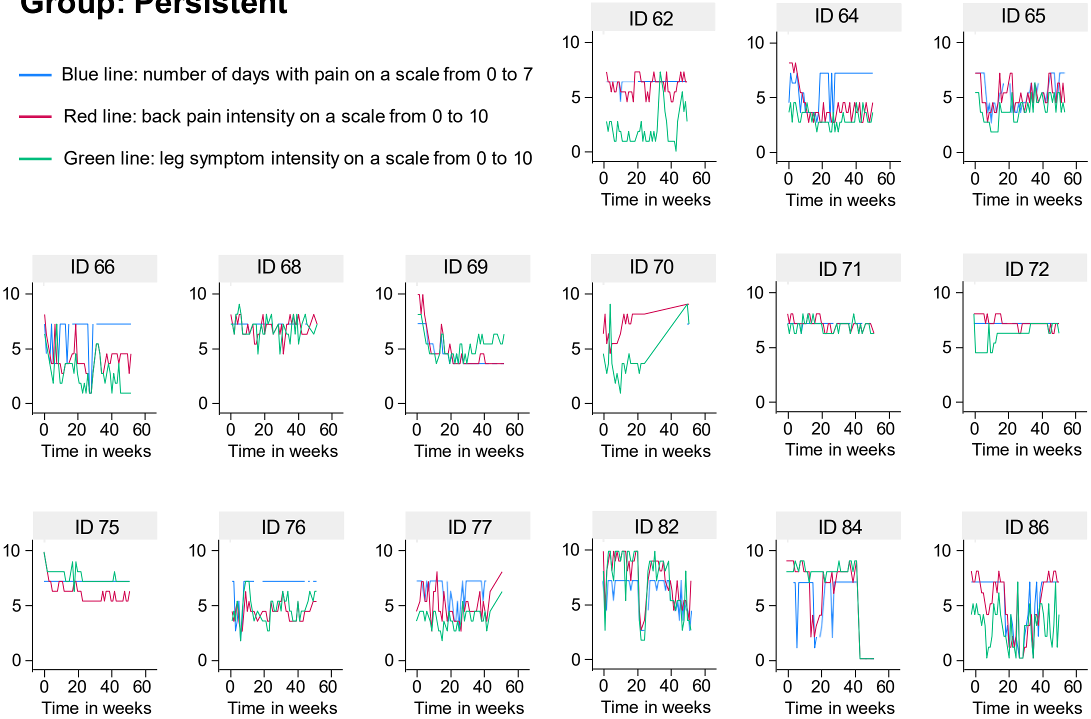

Group: Persistent

Blue line: number of days with pain on a scale from 0 to 7

Red line: back pain intensity on a scale from 0 to 10

Green line: leg symptom intensity on a scale from 0 to 10

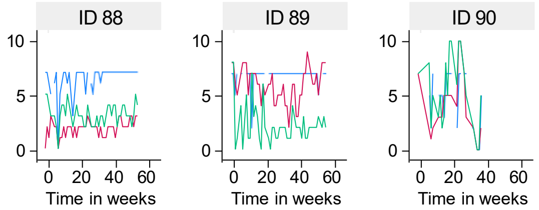

Supplement: Supplementary file 1 — Additional file 1. Individual pain trajectories stratified by trajectory group. [file 13104_2024_6840_MOESM1_ESM.pdf]
